# Structure-Related Electronic and Magnetic Properties in Ultrathin Epitaxial Ni_x_Fe_3−x_O_4_ Films on MgO(001)

**DOI:** 10.3390/nano14080694

**Published:** 2024-04-17

**Authors:** Jari Rodewald, Jannis Thien, Kevin Ruwisch, Tobias Pohlmann, Martin Hoppe, Jan Schmalhorst, Karsten Küpper, Joachim Wollschläger

**Affiliations:** 1 Department of Physics, Osnabrück University, 49076 Osnabrück, Germany; jarodewa@uos.de (J.R.); jthien@uos.de (J.T.); kruwisch@uos.de (K.R.); pohlmann_t@t-online.de (T.P.); martinhoppe@outlook.com (M.H.); kkuepper@uos.de (K.K.); 2 Deutsches Elektronen-Synchrotron (DESY), Photon Science, 22607 Hamburg, Germany; 3 Center for Spinelectronic Materials and Devices, Department of Physics, Bielefeld University, Universitätsstraße 25, 33615 Bielefeld, Germany; jan.schmalhorst@uni-bielefeld.de

**Keywords:** nickel ferrite, ultrathin films, strain-property relation, disordererd phase, synchrotron radiation, X-ray diffraction, hard X-ray photoelectron spectroscopy, X-ray absorption spectroscopy, X-ray circular dichroism, SQUID

## Abstract

Off-stoichiometric Ni_x_Fe_3−x_O_4_ ultrathin films (x < 2.1) with varying Ni content x and thickness 16 (±2) nm were grown on MgO(001) by reactive molecular beam epitaxy. Synchrotron-based high-resolution X-ray diffraction measurements reveal vertical compressive strain for all films, resulting from a lateral pseudomorphic adaption of the film to the substrate lattice without any strain relaxation. Complete crystallinity with smooth interfaces and surfaces is obtained independent of the Ni content x. For x < 1 an expected successive conversion from Fe_3_O_4_ to NiFe_2_O_4_ is observed, whereas local transformation into NiO structures is observed for films with Ni content x > 1. However, angle-resolved hard X-ray photoelectron spectroscopy measurements indicate homogeneous cationic distributions without strictly separated phases independent of the Ni content, while X-ray absorption spectroscopy shows that also for x > 1, not all ${\mathrm{Fe}}^{2+}$ cations are substituted by ${\mathrm{Ni}}^{2+}$ cations. The ferrimagnetic behavior, as observed by superconducting quantum interference device magnetometry, is characterized by decreasing saturation magnetization due to the formation of antiferromagnetic NiO parts.

## 1. Introduction

Transition metal ferrites with a (inverse) spinel structure represent a promising class of oxide-based materials for applications in, for example, spincaloritronics or spintronics [1,2,3,4] due to several intriguing properties, like significant magnetic saturation moments and high Curie temperatures [5]. In the field of spintronics, inverse spinel ferrites like NiFe_2_O_4_ and CoFe_2_O_4_ are suitable for application as a spin filter [6,7,8,9], where highly spin-polarized tunneling currents are generated. The insulating and ferrimagnetic properties of nickel and cobalt ferrite with an exchange splitting of the energy levels in the conduction band result in different tunneling probabilities for the two spin orientations, which make these ferrites highly suited as spin filters [4]. However, the spin-filter efficiency is crucially determined by the structural quality of the tunneling barrier [8] and its interfaces [10]. Thus, ferrites have to be prepared as thin films with low defect densities to obtain a high transmission of spin-polarized tunneling currents.

Here, MgO(001) is well suited as a substrate for NiFe_2_O_4_ films since both materials show a cubic crystalline structure and their lattice mismatch is small. Comparing the lattice constant $a_{{\mathrm{NiFe}}_{2}O_{4}}$ = 833.9 pm of NiFe_2_O_4_ (inverse spinel structure) with twice the lattice constant $a_{\mathrm{MgO}}$ = 421.2 pm of MgO (rocksalt structure), the lattice mismatch is only −1.0%. Hence, only small-strain and low-defect densities are expected when depositing ultrathin NiFe_2_O_4_ films on MgO(001). However, due to the almost doubled ferrite (inverse) spinel lattice constant compared to the one of the rocksalt lattice constant of MgO(001), antiphase boundaries (APBs) are likely to emerge and represent a small drawback in the use of substrates with an almost half-sized lattice constant.

Here, we like to study ultrathin films of Ni ferrite (NFO = Ni_x_Fe_3−x_O_4_) with variable Ni content x. Thus, lattice matching of magnetite Fe_3_O_4_ (NFO with x = 0, inverse spinel) and NiO (NFO in the limit of no Fe content, rocksalt) have to be considered, too. Compared to MgO, the lattice mismatch of Fe_3_O_4_ ($a_{{\mathrm{Fe}}_{3}O_{4}}$ = 839.6 pm) and NiO ($a_{\mathrm{NiO}}$ = 419.5 pm) is only −0.3% and −0.4%, respectively. Thus, it is expected that MgO(001) substrates are also well suited for epitaxy of such oxide films.

For the inverse spinel structure of NiFe_2_O_4_, on the one hand, Ni cations are in oxidation state 2+ and are octahedrally coordinated (occupation of B-sublattice sites). On the other hand, Fe cations are in oxidation state 3+. Half of them are octahedrally coordinated (occupation of B-sublattice sites), while the other half is tetrahedrally coordinated (occupation of A-sublattice sites). Due to the super exchange and double exchange between the different cations, the magnetization is effectively produced by ${\mathrm{Ni}}_{\mathrm{oct}}^{2+}$ cations. Neglecting orbital contributions due to orbital quenching, the magnetic moment of ${\mathrm{Ni}}_{\mathrm{oct}}^{2+}$ is 2 $\mu_{B}$. Similar statements are also valid for Fe_3_O_4_ (magnetite) in which the role of ${\mathrm{Ni}}_{\mathrm{oct}}^{2+}$ cations is adopted by ${\mathrm{Fe}}_{\mathrm{oct}}^{2+}$ cations with magnetic moment of 4 $\mu_{B}$. In NiO, Ni cations are also octahedrally coordinated, but the magnetic order is antiferromagnetic.

Thus, for the Ni ferrite Ni_x_Fe_3−x_O_4_ with varying Ni content x, the chemical composition, i.e., the cationic ratio, as well as lattice site occupation can have a significant impact on their structural, chemical, magnetic, and electronic properties [11,12,13,14]. However, in contrast to investigations focusing on the cationic Co:Fe ratio within Co_x_Fe_3−x_O_4_ films [12,13], a systematic study on the influence of the cationic stoichiometry in Ni_x_Fe_3−x_O_4_ films is still lacking. Therefore, we report on our preparation of Ni_x_Fe_3−x_O_4_ thin films on MgO(001) via reactive molecular beam epitaxy (RMBE) and investigate the influence of the cationic stoichiometry on the structural, chemical, and magnetic properties.

The chemical composition and structure of the Ni_x_Fe_3−x_O_4_ film surfaces were characterized in-situ by means of laboratory-based X-ray photoelectron spectroscopy (XPS) and low-energy electron diffraction (LEED), respectively. Further, the bulk structure was analyzed by synchrotron-based high-resolution X-ray diffraction (HR-XRD), whereas film thicknesses were determined by analysis of X-ray reflectivity (XRR) measurements. The chemical composition and cationic valence states in deeper layers were investigated by hard X-ray photoelectron spectroscopy (HAXPES). In addition, angle-resolved HAXPES (AR-HAXPES) measurements reveal information about the depth-dependent cationic stoichiometry. X-ray absorption spectroscopy (XAS) measurements were complementarily conducted to obtain information about the cationic valence states and site occupancies by comparing the data to charge-transfer multiplet (CTM) calculations. Magnetic properties were studied by means of a superconducting quantum interference device (SQUID).

## 2. Experimental Details

A considerable number of Ni_x_Fe_3−x_O_4_ film with film thicknesses 16 ±2 nm and varying Ni content x (0 ≤ x ≤ 2.07) were prepared on MgO(001) substrates via RMBE in an ultra high vacuum (UHV) chamber. Prior to film growth, the substrates were annealed at 400 °C for 1 h in a molecular oxygen atmosphere of 1 × 10^−4^ mbar to obtain clean and well-ordered substrate surfaces. The chemical purity and surface structure were controlled after substrate annealing by means of laboratory-based in situ XPS and LEED, respectively. Ultrathin Ni_x_Fe_3−x_O_4_ films were deposited on the cleaned substrates via thermal co-evaporation of Ni and Fe via electron bombardment of the respective pure metal rods in a molecular oxygen atmosphere of 5 × 10^−6^ mbar, while the substrate temperature was controlled to 250 °C during film growth. The Ni:Fe ratio in the ferrite films was varied by tuning individually the flux from each evaporation source in a way that the total flux from both sources was kept constant. We obtained a deposition rate of 1.2 (±0.2) nm/min by this procedure. The chemical composition, i.e., the Ni:Fe ratio in the ferrite films as well as the surface structure were again controlled in situ by means of surface sensitive XPS and LEED, respectively. For XPS measurements, Mg $K_{\alpha }$ radiation with a photon energy of $E_{\mathrm{ph}}\left(\mathrm{Mg}\;K_{\alpha }\right)$ = 1253.6 eV was used.

After film preparation and in situ surface characterization (XPS, LEED), film thicknesses were determined ex situ by means of X-ray reflectivity (XRR) in a Philips X’Pert Pro diffractometer at Bielefeld University. Here, Cu $K_{\alpha }$ radiation with a photon energy of $E_{\mathrm{ph}}\left(\mathrm{Cu}\;K_{\alpha }\right)$ = 8048.0 eV was used. XRR data were analyzed using an in-house-developed fitting tool, which is based on the Parratt algorithm [15] and the Névot–Croce roughness model [16].

Additionally, the ferrite ultrathin film structure was characterized by high resolution X-ray diffraction (HR-XRD) measurements at beamline P08 of PETRA III at the Deutsches Elektronen-Synchrotron (DESY), Germany. Here, a photon energy of $E_{\mathrm{ph}}$ = 18 keV was used. The sample and detector were positioned with respect to the incident beam by using a six-circle (4S+2D-type) diffractometer (KOHZU NZD-3), while data acquisition was conducted by using a two-dimensional detector (PILATUS 100k or DECTRIS EIGER 1M). The HR-XRD measurements were conducted in θ-2θ geometry to perform out-of-plane scans along the (00L) crystal truncation rod (CTR) across the MgO(002) and NFO(004) Bragg reflections. Analysis of HR-XRD data was performed by using an in-house-developed fitting tool, which employs full kinematic diffraction theory.

Complementary to laboratory-based surface sensitive XPS, HAXPES measurements were conducted at beamline P09 of PETRA III at DESY. In contrast to XPS, the higher used photon energy of $E_{\mathrm{ph}}$ = 6 keV for HAXPES measurements excites photoelectrons with higher kinetic energy and, thus, higher inelastic mean free paths (IMFPs) λ and consequently higher information depths $D_{I}$. Thus, HAXPES is used to determine the chemical composition and cationic valencies not only in the near-surface region (as probed by XPS) but also in deeper subsurface layers. The endstation is equipped with a SPECS Phoibos 225 HV hemispherical analyzer with a delay line detector to record HAXPES spectra at beamline P09.

Additionally, a wide-angle lens with ±30° angular acceptance was used to record angle-resolved HAXPES (AR-HAXPES) spectra of photoelectrons with different off-normal emission angles ϕ. The angular-dependent information depth $D_{I}^{95}$, from which 95% of photoelectrons (detected at the off-normal emission angle ϕ) originate, is given by

(1) $$D_{I}^{95}\left(\phi \right)≃3\lambda \cos\phi$$

with λ as the IMFP of the respective photoelectrons as determined by the Tanuma, Powell, and Penn (TPP-2M) algorithm [17]. As a consequence, varying the detection angle ϕ in AR-HAXPES measurements allows for depth-dependent photoelectron detection (cf. Table 1). For this purpose, the incident angle $\theta =40^{\circ }$ between the incident beam and surface plane was kept constant, and the data detected within the acceptance angle sections of $8^{\circ }$ were integrated for better statistics.

Complementarily, XAS measurements were performed to probe the cationic valence states and the lattice site occupancies within the film lattice. These measurements were performed at the Superconducting Vector Magnet Endstation at beamline 4.0.2 of the Advanced Light Source (ALS). All samples were measured at room temperature with an incident angle of $30^{\circ }$ towards the [100] direction of the ferrite films. The XAS spectra were measured across the Fe $L_{2,3}$ (690–750 eV) and Ni $L_{2,3}$ (835–890 eV) absorption edges in the total electron yield (TEY) mode, which is surface sensitive with a probing depth of ∼5 nm in, e.g., Fe_3_O_4_ [18,19].

In order to quantify the cationic lattice site occupancies in the ferrite film, XAS measurements were analyzed by comparing the data to spectra obtained by charge transfer multiplet (CTM) calculations. In these calculations, a molecule complex composed of the respective cation in a given ligand crystal field is taken as a basis, and charge transfer is considered between the cation and ligand. The total CTM calculated spectrum is obtained by a weighted linear superposition of the individual spectra of each cation. By comparing the calculation with the experimental XAS spectra, quantitative information about the site occupancies of each cation is determined. In addition to crystal field effects and the charge transfer interaction, the CTM models include 100% spin–orbit coupling. Further, the Slater integrals F(dd), F(pd), and G(pd), which account for *d*-*d* and *p*-*d* Coulomb and exchange interactions [20], were each reduced to 80%, which is consistent within ±10% with previous studies on Fe_3_O_4_ [21,22,23,24,25]. Charge transfer is considered by setting the O 2*p*-Fe 3*d* charge transfer energies $\Delta_{\mathrm{init}}$ and $\Delta_{\mathrm{final}}$ of the initial and final states, respectively. The best results for these parameters as well as for the crystal field energies 10Dq were obtained for the values listed in Table 2 for the respective cations in a given ligand field, which are similar to the values used in former studies on Fe_3_O_4_ [25,26].

Additionally, the CTM calculated spectra were compared to the data by assuming a Gaussian instrumental broadening of 0.25 eV and a Lorentzian lifetime broadening of 0.3 eV (0.6 eV) for $L_{3}$ ($L_{2}$) edges.

Magnetic properties of the Ni_x_Fe_3−x_O_4_ films were characterized by using a SQUID of type S700X from CRYOGENIC. Here, the magnetization *M* was recorded at room temperature as a function of applied magnetic field *H*, which was tuned from +7 T to −7 T and back again to obtain *M* vs. *H* curves, which show hysteretic behavior for ferrimagnetic or ferromagnetic material.

## 3. Results

### 3.1. Surface Characterization: XPS and LEED

The chemical composition, i.e., the ratio between Ni and Fe of the Ni_x_Fe_3−x_O_4_ films, was determined by analyzing the Ni 3*p* and Fe 3*p* spectra measured in situ by surface sensitive XPS.

**Figure 1:**
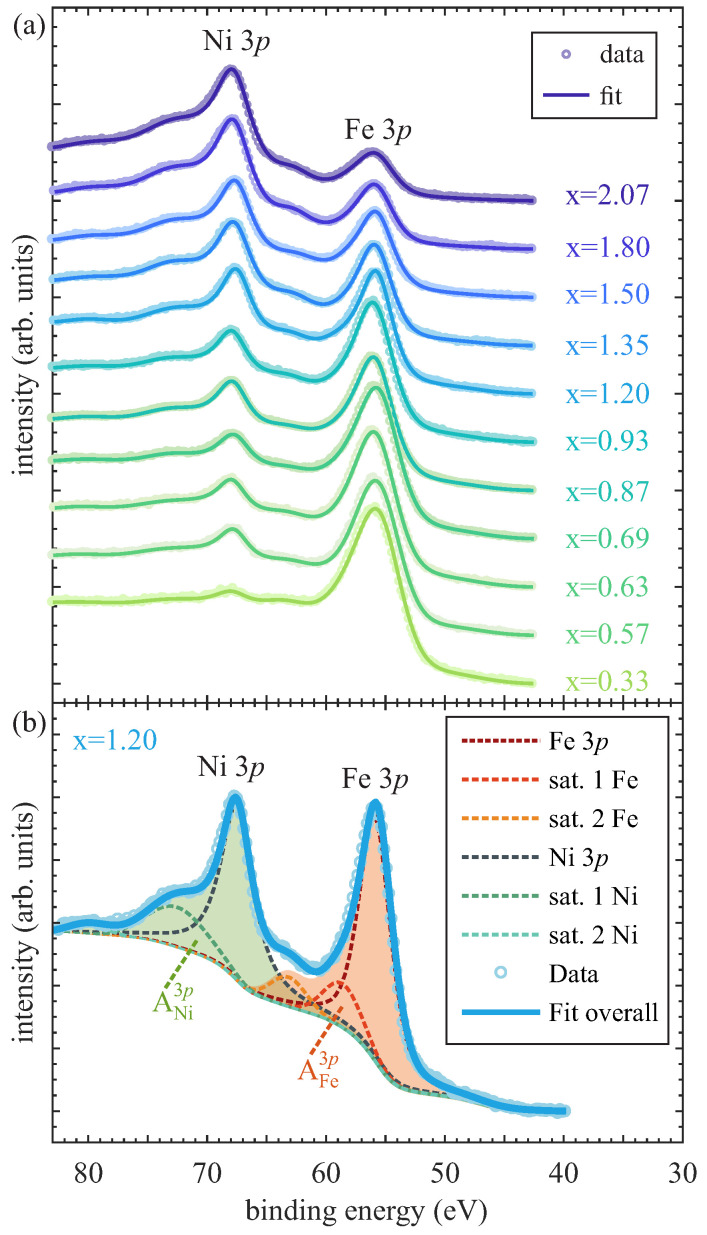
(**a**) Ni 3*p* and Fe 3*p* spectra of Ni_x_Fe_3−x_O_4_ ultrathin films with varying Ni amount x measured by XPS. (**b**) Exemplary analysis of both Ni 3*p* and Fe 3*p* spectra for Ni content x = 1.20. Both main lines are accompanied by two satellites at their high binding energy sides. The Ni content x is determined by taking the background subtracted areas $A_{\mathrm{Fe}}^{3p}$ and $A_{\mathrm{Ni}}^{3p}$ (filled regions) into account.

The corresponding spectra are depicted in Figure 1a. We assumed that the O 1 s photoelectron peaks are located at 530 eV binding energy to calibrate the energy scale of all spectra. For increasing Ni content x within the ferrite films, the intensity of the Ni 3*p* spectrum increases as compared to the decreasing Fe 3*p* intensity. To determine the Ni:Fe ratio, the areas below the Ni 3*p* and Fe 3*p* spectra were determined after subtracting a Shirley background and deconvoluting each 3*p* spectrum into one main 3*p* photoemission line and two satellites located at the higher binding energy sides of each 3*p* spectrum [cf. Figure 1b]. The main photoemission lines and satellites were each fitted by a convolution of a Lorentzian and a Gaussian. The sum of all fitted peaks results in the overall fits, which completely match with the measured data of each ferrite film, is shown in Figure 1a. As the binding energies of Ni 3*p* and Fe 3*p* spectra only differ by a few eV (∼12 eV) from each other, the energy-dependent IMFPs $\lambda_{\mathrm{Ni}}^{3p}\approx \lambda_{\mathrm{Fe}}^{3p}$ = 2.3 nm ($D_{I}^{95}$ = 6.9 nm) and the transmission function of the spectrometer are also very similar for these photoelectrons. As a consequence, the Ni content $x\;=\;3\cdot Y_{\mathrm{Ni}}^{3p}$ of the prepared ferrite films is determined via the relative photoelectron yield

(2) $$Y_{\mathrm{Ni}}^{3p}=\frac{I_{\mathrm{Ni}}^{3p}}{I_{\mathrm{Ni}}^{3p}+I_{\mathrm{Fe}}^{3p}}=\frac{A_{\mathrm{Ni}}^{3p}/\sigma_{\mathrm{Ni}}^{3p}}{A_{\mathrm{Ni}}^{3p}/\sigma_{\mathrm{Ni}}^{3p}+A_{\mathrm{Fe}}^{3p}/\sigma_{\mathrm{Fe}}^{3p}},$$

with $A_{\mathrm{Ni},\mathrm{Fe}}^{3p}$ as the areas below the background subtracted Ni and Fe 3*p* spectra [including satellites, cf. Figure 1b] and $\sigma_{\mathrm{Ni},\mathrm{Fe}}^{3p}$ as the respective photoelectric cross sections [27]. The resulting Ni content x for the presented XPS spectra are given next to the respective measurements in Figure 1a. However, due to the variable contributions of the satellites in the 3*p* spectra, an experimental error of Δx = ±0.08 has to be considered.

**Figure 2:**
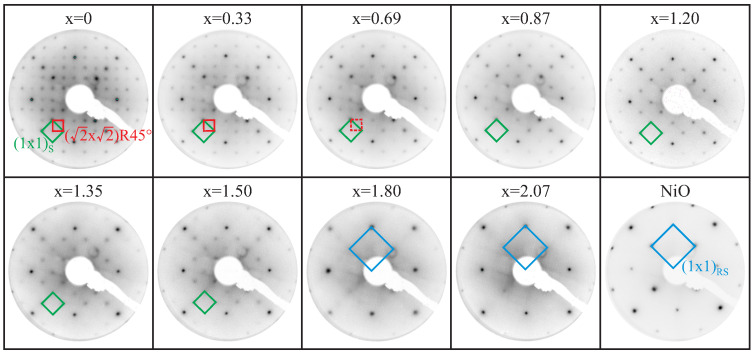
LEED pattern at an electron energy of 162 eV for several exemplary Ni_x_Fe_3−x_O_4_ ultrathin films on MgO(001) with varying Ni content x. The $\left(\sqrt{2}\times \sqrt{2}\right)R45^{\circ }$ superstructure (red square), characteristic for an Fe_3_O_4_(001) surface (x = 0), vanishes for Ni contents of x ≥ 0.69, whereas the spinel type ${\left(1\times 1\right)}_{S}$ surface structure (green square) stays visible up to x = 1.50. For x ≥ 1.80 solely, a square ${\left(1\times 1\right)}_{\mathrm{RS}}$ structure (blue square) is obtained, which exhibits twice-as-large reciprocal lattice constants than the reciprocal unit cell of the spinel type ${\left(1\times 1\right)}_{S}$ structure and corresponds to the rock-salt-type surface unit cell as seen by comparison to a LEED pattern recorded from a NiO film.

In addition to the determination of the chemical composition by means of surface sensitive XPS, the surface structure of the prepared ferrite films is determined via in situ LEED measurements. Figure 2 shows an exemplary selection of the representative LEED pattern for varying Ni amounts in the ferrite films. For better comparison, the electron energy of 162 eV is the same for all depicted diffraction patterns.

For the Fe_3_O_4_ film (x = 0), a clear $\left(\sqrt{2}\times \sqrt{2}\right)R45^{\circ }$ superstructure in addition to the spinel type (index S) square ${\left(1\times 1\right)}_{S}$ surface structure is visible. This superstructure is characteristic for Fe_3_O_4_(001) surfaces and vanishes for Ni contents x ≥ 0.69, whereas the spinel-type ${\left(1\times 1\right)}_{S}$ surface structure stays visible up to x = 1.50. For even larger Ni contents x ≥ 1.80, solely a square ${\left(1\times 1\right)}_{\mathrm{RS}}$ surface structure is visible, which exhibits reciprocal surface lattice constants that are twice as large as the spinel-type reciprocal lattice constants in the ${\left(1\times 1\right)}_{S}$ unit cell. This larger reciprocal unit cell corresponds to the surface unit cell of a rock salt type surface (index RS), as it is obtained for LEED at, for example, NiO surfaces.

These observations indicate the formation of merely spinel-type structures for low Ni content x, while the sole presence of a ${\left(1\times 1\right)}_{\mathrm{RS}}$ surface structure as obtained for x ≥ 1.80 with twice as large reciprocal surface lattice vectors (meaning half the surface lattice vectors in real space) compared to the unit cell of the spinel-type ${\left(1\times 1\right)}_{S}$ structure points to a major formation of rock-salt-type structures at the surface. However, the coexistence of a spinel structure and rock salt structure with a superposed diffraction pattern is assumed for intermediate Ni content x. Here, we like to emphasize that the deviation between the LEED reflex intensities for the film with x = 2.07 compared to the reflex intensities obtained for the NiO surface (although the electron energy is the same in both measurements) indicates a different rock-salt-type structure, e.g., an Fe-doped NiO phase, pointing at the surface for x = 2.07.

### 3.2. XRR

**Figure 3:**
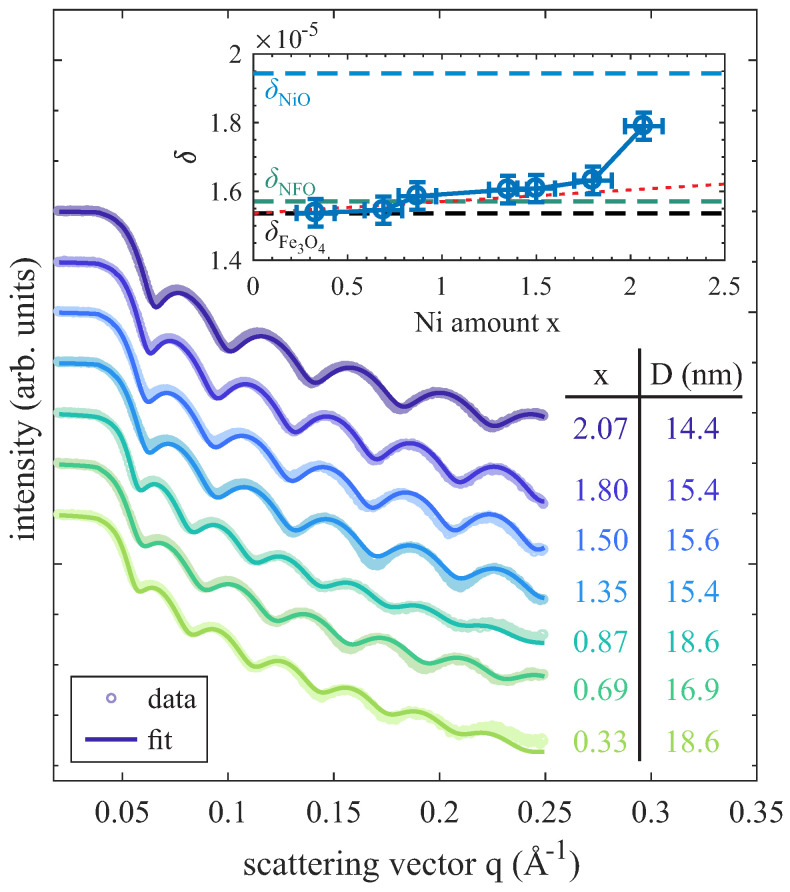
XRR measurements for Ni_x_Fe_3−x_O_4_ ultrathin films of film thickness D with varying Ni content x. The calculated XRR curves are in excellent agreement with the measured data. Clear Kiessig fringes point to low surface and interface roughness for all films. The dispersion δ is increasing for increasing Ni amount x (shown in the inset). The dispersion values for stoichiometric Fe_3_O_4_, NiFe_2_O_4_ (NFO), and NiO at the used X-ray energy of 8048.0 eV (Cu Kα) are depicted for comparison (dashed lines). The red dotted line represents the (linear) evolution of δ if Ni cations were solely incorporated on spinel-type lattice sites with increasing x.

In addition to the in situ surface characterization by means of XPS and LEED, XRR measurements were conducted ex situ after transport under ambient conditions. Representative measurements of some Ni_x_Fe_3−x_O_4_ films with varying Ni content x and the corresponding calculated XRR curves are shown in Figure 3. XRR data for all films show clear Kiessig fringes. They result from the interference of X-rays reflected at the film surface and at the interface between the film and substrate and point to the low interface and surface roughness independent of the Ni content x. For the detailed analysis of XRR curves, the refractive index $\delta_{\mathrm{MgO}}$ of the MgO substrate at the used X-ray energy of $E_{\mathrm{ph}}\left(\mathrm{Cu}\;K_{\alpha }\right)$ = 8048.0 eV was kept fixed [28]. In contrast, the thickness, interface/surface roughness, and refractive index of the film were used as free fit parameters. As seen in Figure 3, the calculated XRR curves are in excellent agreement with the measured data, pointing to single homogeneous films for all Ni contents x. The resulting film thicknesses range from 14.0 to 18.6 nm. More remarkably, the obtained dispersions δ ($\delta \propto \rho_{\mathrm{el}}$ with $\rho_{\mathrm{el}}$ as the electron density) are increasing for an increasing Ni amount x in the ferrite film (cf. inset of Figure 3), as it is expected considering the literature values of stoichiometric Fe_3_O_4_ ($\delta_{{\mathrm{Fe}}_{3}O_{4}}\;=\;1.54\cdot 10^{-5}$), NiFe_2_O_4_ ($\delta_{\mathrm{NFO}}\;=\;1.57\cdot 10^{-5}$), and NiO ($\delta_{\mathrm{NiO}}\;=\;1.94\cdot 10^{-5}$) [28]. For Ni amounts x ≤ 1.50, only a slight increase in δ is observed with increasing Ni content x, matching the linear evolution obtained theoretically if Ni and Fe cations solely occupy spinel-type lattice sites (red dotted line) according to the slight increase in the literature values of Fe_3_O_4_ and NiFe_2_O_4_. In contrast, for x ≥ 1.80 (in particular for x = 2.07), the dispersions are clearly exceeding these values and are approaching the dispersion expected for NiO, which enforces the assumption that spinel ${\mathrm{NiFe}}_{2}$O_4_ coexists with rock salt NiO (probably with Fe doping) and that the NiO fraction increases Ni content x.

### 3.3. HR-XRD

**Figure 4:**
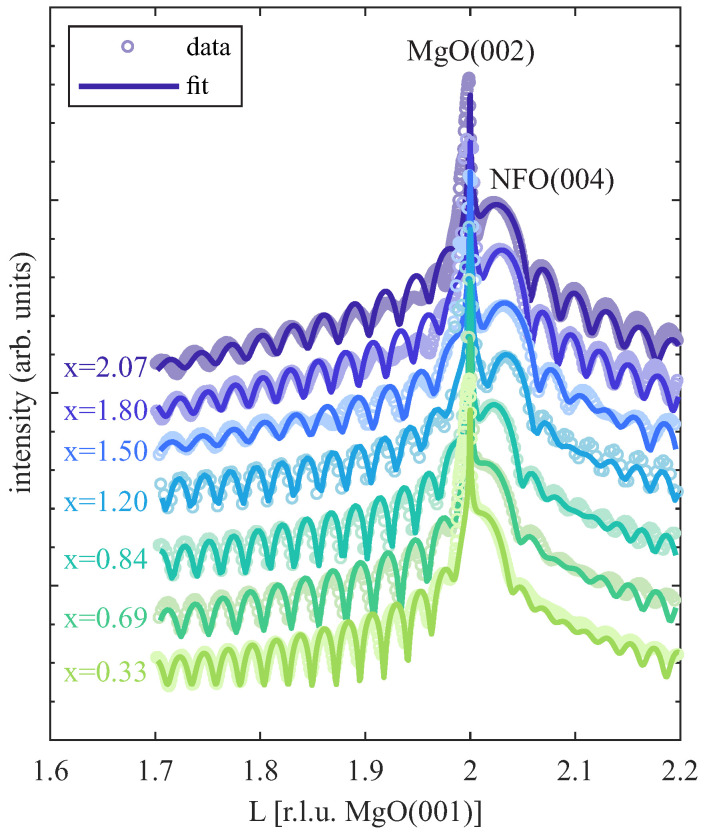
HR-XRD out-of-plane measurements along the (00L) CTR across the MgO(002) and NFO(004) Bragg conditions with corresponding calculations based on full kinematic diffraction theory for NFO films with different Ni content x. The calculated diffractograms show very good agreement with the measured data. Clear Laue fringes point to smooth interfaces and high crystalline quality.

HR-XRD measurements were performed ex situ at the PETRA III P08 beamline at DESY to characterize the structure of the NFO films. The recorded data along the (00*L*) CTR close to the MgO(002) Bragg condition for several representative NFO films with varying Ni amount are depicted in Figure 4. For all measurements, a clear NFO(004) Bragg peak is visible, which is located at slightly higher *L* values than for the MgO(002) reflection due to the slightly lower layer distance of the ferrite film compared to the layer distance of the MgO substrate. Moreover, the measured (00*L*) CTRs exhibit clear Laue fringes, pointing to smooth interfaces and high crystalline film quality, which is in accordance with the XRR results.

**Figure 5:**
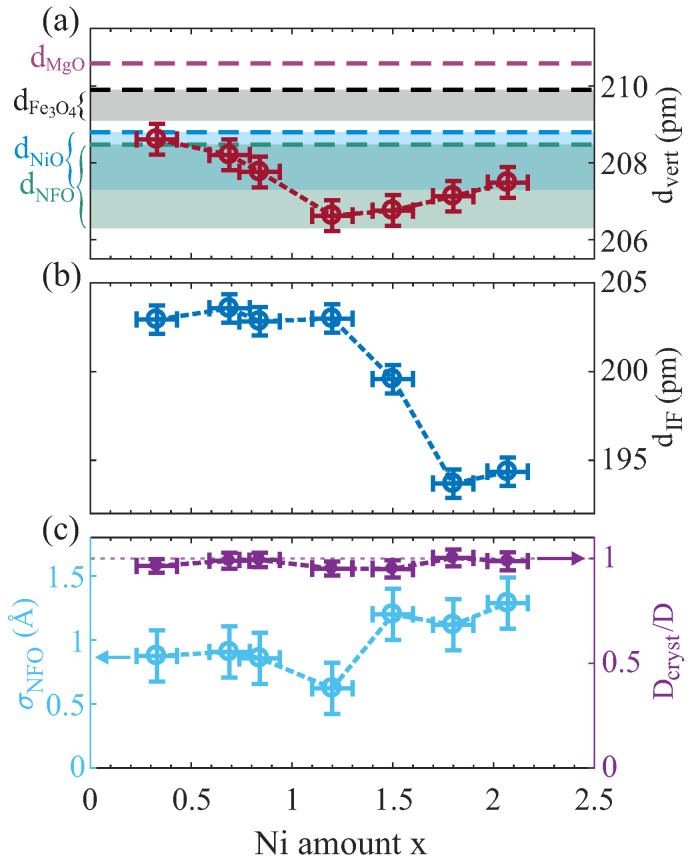
(**a**) Vertical layer distance $d_{\mathrm{vert}}$ compared to bulk layer distances of MgO, Fe_3_O_4_, NiFe_2_O_4_, and NiO (dashed horizontal lines). The filled regions cover the range between completely relaxed (upper boundary) and fully compressed layer distances due to pseudomorphic growth (lower boundary). (**b**) Interface distance $d_{\mathrm{IF}}$, and (**c**) film surface roughness $\sigma_{\mathrm{NFO}}$ and scaled crystalline film thickness $D_{\mathrm{cryst}}$/D as resulting from the calculated models for varying Ni content x.

Further, the recorded HR-XRD data were analyzed using calculations based on full kinematic diffraction theory. The resulting models show very good agreement with the measured diffraction data as shown in Figure 4. From these calculations, parameters like the vertical layer distance $d_{\mathrm{vert}}$, the interface distance $d_{\mathrm{IF}}$, and the number of film monolayers (ML) $N_{\mathrm{ML}}$, as well as the ferrite film surface roughness $\sigma_{\mathrm{NFO}}$, are extracted. From the number of monolayers $N_{\mathrm{ML}}$ and the vertical layer distance $d_{\mathrm{vert}}$, the crystalline film thickness $D_{\mathrm{cryst}}$ = $N_{\mathrm{ML}}$$d_{\mathrm{vert}}$ can be determined and compared to the total film thickness D (as determined by XRR; see above). The resulting values of $d_{\mathrm{vert}}$ and $d_{\mathrm{IF}}$, as well as $\sigma_{\mathrm{NFO}}$ and the scaled crystalline film thickness $D_{\mathrm{cryst}}$/D independent of the Ni amount x within the ferrite films, are depicted in Figure 5a–c, respectively.

The vertical layer spacing $d_{\mathrm{vert}}$ within the NFO films decreases up to a Ni content of x = 1.20 and increases for higher Ni content up to x = 2.07 [see Figure 5a], which can be followed by tracing the position of the NFO(004) Bragg peak, which is increasing in *L* up to x = 1.20 and decreasing again for higher x (see Figure 4), meaning decreasing/increasing layer distances, respectively. This behavior can be understood qualitatively by comparing this trend with the bulk layer distances of stoichiometric Fe_3_O_4_, NiFe_2_O_4_, and NiO [cf. dashed lines within Figure 5a]. Here, Fe_3_O_4_ (x = 0) exhibits the largest layer distance of $d_{{\mathrm{Fe}}_{3}O_{4}}$ = 209.9 pm, whereas for stoichiometric NiFe_2_O_4_ (x = 1), the smallest bulk layer distance of $d_{{\mathrm{NiFe}}_{2}O_{4}}$ = 208.5 pm is obtained, which can explain the decrease in $d_{\mathrm{vert}}$ for increasing x between 0 ≤ x ≤ 1.2. In contrast, bulk NiO exhibits again a slightly larger layer distance of $d_{\mathrm{NiO}}$ = 208.8 pm compared to NiFe_2_O_4_. Thus, a further increase in the Ni amount above the stoichiometric value of x = 1 should also result in an increase in the layer distance $d_{\mathrm{vert}}$ if further Ni cations result in (Fe-doped) NiO-type formations. In fact, this behavior is observed here for x > 1.20 and is therefore consistent with the obtained results if saturation of the spinel-type lattice sites is assumed for x ≥ 1.20. In accordance with the LEED and XRR results, such a saturation of the spinel-type lattice sites can be assumed for the range 1.20 ≤ x ≤ 1.50.

However, as seen in Figure 5a, all films are compressively strained in the vertical direction since the absolute values of $d_{\mathrm{vert}}$ are clearly below the bulk values of Fe_3_O_4_, NiFe_2_O_4_, and NiO for stoichiometries of x < 1, x ≈ 1, and x > 1, respectively. This observation can be understood by assuming a lateral adaption of the ferrite film to the substrate lattice (pseudomorphic growth) and, consequently, the presence of lateral tensile strain, which results in vertical compression within the ferrite films. A more detailed (quantitative) analysis of this assumption is given in Section 4.

Furthermore, the interface distance $d_{\mathrm{IF}}$ is smaller than the determined layer distances and almost constant for Ni amounts x ≤ 1.20, whereas it is significantly decreasing when further increasing the Ni content [see Figure 5b]. Complementary to the LEED results (cf. Figure 2), which indicate the emergence of an almost single rock-salt-like structure at the surface for x > 1.50, the observed trend of the interface distance also points to the formation of another structural phase (at the interface) for a Ni content 1.20 ≤ x ≤ 1.50.

The film surface roughness $\sigma_{\mathrm{NFO}}$, as determined from HR-XRD analysis [cf. Figure 5c], is very low ($\sigma_{\mathrm{NFO}}<1.0$ Å) and constant for x ≤ 1.20, whereas it is only slightly increased but constant for x ≥ 1.50 ($\sigma_{\mathrm{NFO}}\approx 1.3$ Å). In fact, these very low values for all films indicate smooth surfaces, which is in accordance with the observations in XRR and the sharp reflexes obtained in the LEED pattern (see Figure 2), and comes along with complete crystallinity $D_{\mathrm{cryst}}$ = D independent of the Ni content x [cf. Figure 5c].

### 3.4. HAXPES

Complementary to surface sensitive XPS, HAXPES measurements were conducted at P09 of PETRA III at DESY to determine the chemical composition and the cationic valencies also in deeper subsurface layers. While for laboratory based (soft) XPS the maximum information depth $D_{I}^{95}$(ϕ = 0) [cf. Equation (1)] of the analyzed Ni 2*p* and Fe 2*p* photoelectrons passing through NiFe_2_O_4_ is about 3–4 nm, it is crucially enhanced to about 22 nm for HAXPES with an X-ray energy of 6 keV. Hence, the performed HAXPES measurements completely probe the prepared NFO films in vertical direction, as the thicknesses of all films are still well below the maximum information depths. This determination is supported by the fact that the Mg 1*s* photoemission signal originating from the substrate is still visible in all HAXPES measurements.

**Figure 6:**
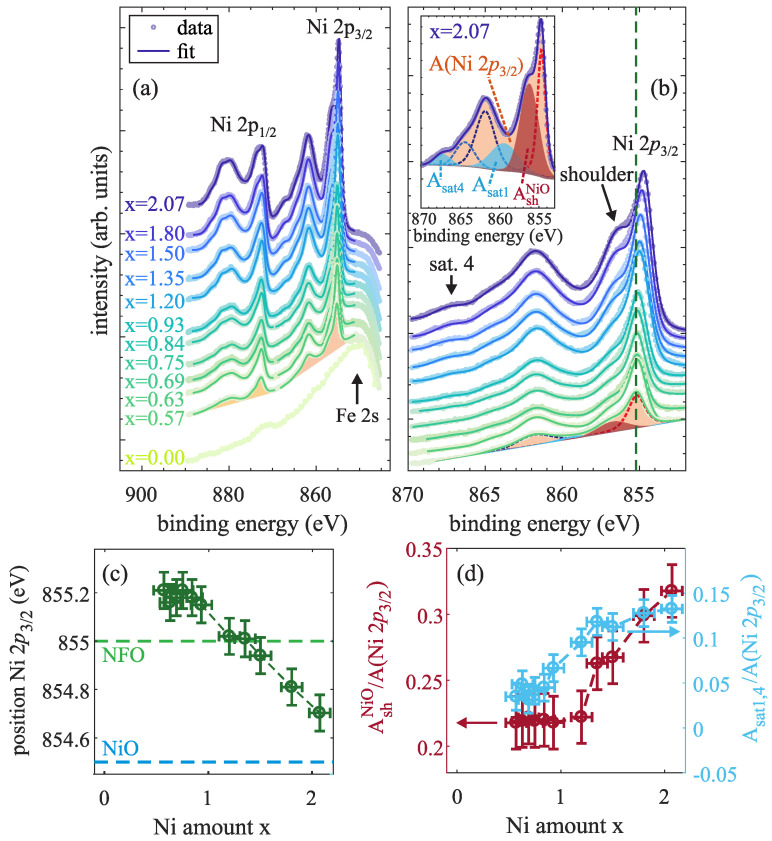
(**a**) Ni 2*p* spectra of several prepared Ni_x_Fe_3−x_O_4_ films with varying Ni content x measured by HAXPES along with overall fits (solid lines). (**b**) Enlarged section of the Ni 2$p_{3/2}$ region with an emerging high binding energy shoulder of the Ni 2$p_{3/2}$ main line for increasing Ni content x. The deconvoluted peaks for the overall fits are depicted in the inset exemplarily for x = 2.07. (**c**) Position of the maximum intensity of the Ni 2$p_{3/2}$ main line. (**d**) Background subtracted areas of the high binding energy shoulder $A_{\mathrm{sh}}^{\mathrm{NiO}}$ (red circles) and of the emerging satellites $A_{\mathrm{sat}1,4}$ = $A_{\mathrm{sat}1}$+$A_{\mathrm{sat}4}$ (blue circles) both compared to the area below the entire Ni 2$p_{3/2}$ spectrum A(Ni 2$p_{3/2}$).

The recorded Ni 2*p* spectra for Ni_x_Fe_3−x_O_4_ films with varying Ni content x are depicted in Figure 6a. All spectra exhibit the main spin–orbit split 2$p_{1/2}$ and 2$p_{3/2}$ photoemission peaks accompanied by satellites with ∼7 eV larger apparent binding energies. These spectra are characteristic for ${\mathrm{Ni}}^{2+}$ present in several oxides as, e.g., NiO or NiFe_2_O_4_ [29,30,31].

For a more detailed analysis, the Ni 2$p_{3/2}$ spectra were fitted by several peaks [cf. inset of Figure 6b], each one described by a convolution of a Lorentzian and a Gaussian. The number and positions of individual peaks were based on the theoretical description of Ni 2*p* spectra by Veenendaal and Sawatzky [31], resulting in one peak for the main photoemission line in addition to one peak describing the high binding energy shoulder, as well as four contributions to the satellite structure located ∼7 eV above the main Ni 2$p_{3/2}$ line [cf. Figure 6b].

As seen in Figure 6a,b, the Ni 2*p* spectra undergo several changes when varying the Ni content x within the ferrite films. First, the binding energy position of the Ni 2$p_{3/2}$ main photoemission line (determined as the position of the maximum overall intensity) for Ni contents x < 1.0 remains constant at ∼855.2(1) eV, while it is significantly decreasing for further increasing Ni content x > 1.0 [cf. Figure 6b,c]. For Ni contents x close to the stoichiometric NiFe_2_O_4_ value of x = 1 the Ni 2$p_{3/2}$ position is comparable to the value of 855.0(2) eV for Ni 2$p_{3/2}$ in NiFe_2_O_4_ as reported by Kuschel et al. [32]. Since the Ni 2$p_{3/2}$ position of ∼854.5 eV as reported for NiO [33] is significantly lower than the Ni 2$p_{3/2}$ position in NiFe_2_O_4_, the observed subsequent decrease in the binding energy down to ∼854.7 eV for x = 2.07 is a behavior to be expected if the NiO content of the oxide film increases with increasing the Ni content x.

In addition to the decreasing Ni 2$p_{3/2}$ position, the high binding energy shoulder ∼1.5 eV apart from the main line is significantly enhanced [and visibly distinguishable from the main line, cf. Figure 6b] for x > 1.35 compared to lower Ni contents x. In particular, the background subtracted area $A_{\mathrm{sh}}^{\mathrm{NiO}}$ of this shoulder in relation to the total area A(Ni 2$p_{3/2}$) of the Ni 2$p_{3/2}$ spectrum is steadily increasing for increasing Ni content x ≥ 1.35 [cf. Figure 6d]. This shoulder can be theoretically described as a result of a screening process by electrons not originating from the oxygen orbitals around the respective Ni atom, but from neighboring ${\mathrm{NiO}}_{6}$ clusters (with ${\mathrm{Ni}}^{2+}$ cations occupying octahedral sites) [31]. Hence, the appearance and enhancement of this shoulder can be ascribed to the presence and increasing content of NiO within the ferrite films with increasing x.

Furthermore, the satellite structure ∼7 eV above the main Ni 2$p_{3/2}$ line slightly changes during variation of the Ni content x. In particular, the intensities of the two satellites at ∼859.2 (sat. 1) and ∼866.9 eV [sat. 4, cf. inset of Figure 6b] change for varying Ni content x. Figure 6d shows the summed up intensities areas $A_{\mathrm{sat}1,4}$ = $A_{\mathrm{sat}1}$ + $A_{\mathrm{sat}4}$ (after background subtraction) in relation to the overall intensity A(Ni 2$p_{3/2}$) of the Ni 2$p_{3/2}$ spectrum. While the combined intensity of both satellites almost vanishes for low Ni contents x < 1.0, it increases with further increasing x above the stoichiometric value (x > 1.0). According to Veenendaal and Sawatzky, these satellites also result from the presence of neighboring ${\mathrm{NiO}}_{6}$ clusters. Hence, the observed increasing intensities of both high binding energy shoulder and the mentioned satellites correspond to the enhanced formation of NiO within the ferrite films with increasing Ni amount x.

**Figure 7:**
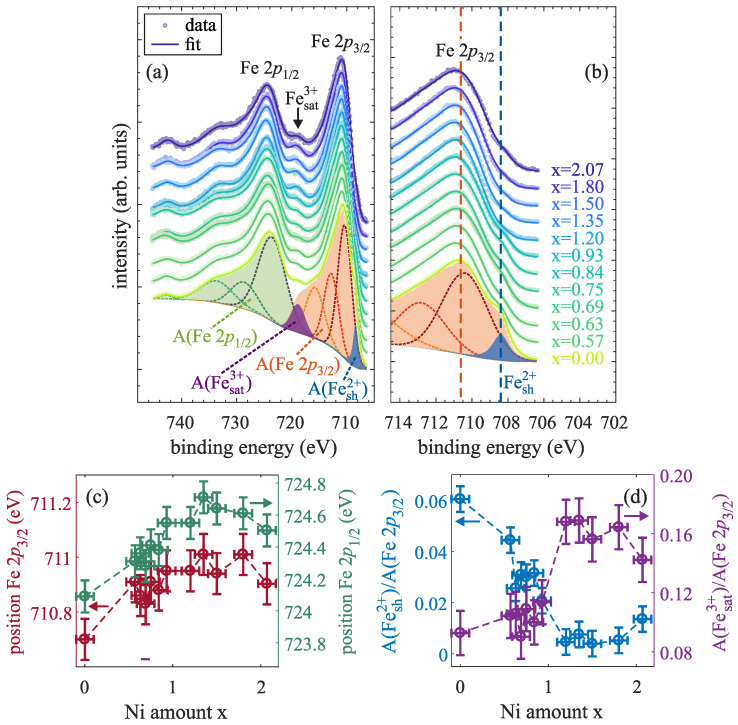
(**a**) Fe 2*p* spectra of several prepared Ni_x_Fe_3−x_O_4_ films with varying Ni content x measured by HAXPES along with overall fits (solid lines). The deconvoluted peaks for the overall fit are depicted exemplarily for the Fe_3_O_4_ film (x = 0) with filled background subtracted areas of the Fe 2$p_{3/2}$ (red) and the Fe 2$p_{1/2}$ spectrum (green). (**b**) Enlarged section of the Fe 2$p_{3/2}$ region with a low binding energy shoulder ${\mathrm{Fe}}_{\mathrm{sh}}^{2+}$ of the Fe 2$p_{3/2}$ main line with slightly increasing binding energy from 710.7 eV for x = 0 to 711.0 eV for x ≥ 1 (see below). Additionally, the position of the Fe 2$p_{3/2}$ maximum as reported in literature for the case of Fe_3_O_4_ is marked by a (red) dashed line. (**c**) Positions of the maximum intensity of the Fe 2$p_{3/2}$ and Fe 2$p_{1/2}$ main lines. (**d**) Background subtracted areas of the low binding energy shoulder A(${\mathrm{Fe}}_{\mathrm{sh}}^{2+}$) and of the ${\mathrm{Fe}}^{3+}$ satellite A(${\mathrm{Fe}}_{\mathrm{sat}}^{3+}$) compared to the area below the whole Fe 2$p_{3/2}$ spectrum A(Fe 2$p_{3/2}$).

In addition, Fe 2*p* HAXPES spectra were recorded to determine the existent valence states of Fe cations within the ferrite films with varying Ni:Fe ratios, as depicted in Figure 7a. For analysis, both spin–orbit split Fe 2$p_{3/2}$ and Fe 2$p_{1/2}$ background subtracted spectra were fitted by several functions, which are each described by a convolution of a Lorentzian and a Gaussian. Also here, the Fe 2$p_{3/2}$ region is analyzed in more detail. It generally consists of two peaks forming the main photoemission line as well as of two charge-transfer satellites at ∼715.6 and ∼718.8 eV, which can be assigned to the presence of ${\mathrm{Fe}}^{2+}$ and ${\mathrm{Fe}}^{3+}$, respectively [34]. Moreover, a shoulder ${\mathrm{Fe}}_{\mathrm{sh}}^{2+}$ at the low binding energy side of the main line at ∼708.4 eV [cf. enlarged region of the Fe 2$p_{3/2}$ region in Figure 7b] results from the presence of ${\mathrm{Fe}}^{2+}$ cations [30].

First, the binding energies of the main Fe 2$p_{3/2}$ and Fe 2$p_{1/2}$ lines can serve as an indication for the major Fe valency. Both values were determined as the positions of the maximum intensity of the overall fit in each region and are depicted in Figure 7c in dependence of the Ni amount x. For the Fe_3_O_4_ film (x = 0), binding energies of 710.7(1) and 724.1(1) eV are obtained for the Fe 2$p_{3/2}$ and Fe 2$p_{1/2}$ line, respectively, which both are in accordance with the values of stoichiometric Fe_3_O_4_ reported in literature [34]. Both positions shift to higher values for increasing Ni content in the regime x < 1. If x is further increased (x > 1), both Fe 2$p_{3/2}$ and Fe 2$p_{1/2}$ positions approximately remain constant at 711.0(1) and 724.6(1) eV, respectively, which agree with reported values obtained for Fe_2_O_3_, where only ${\mathrm{Fe}}^{3+}$ cations are present [34]. Thus, it seems that there are no ${\mathrm{Fe}}^{2+}$ cations but solely ${\mathrm{Fe}}^{3+}$ cations in the oxide film.

Second, the intensity peak areas A(${\mathrm{Fe}}_{\mathrm{sat}}^{3+}$) and A(${\mathrm{Fe}}_{\mathrm{sh}}^{2+}$) (after background subtraction) of the respective ${\mathrm{Fe}}_{\mathrm{sat}}^{3+}$ charge-transfer satellite at ∼718.8 eV and the low binding energy shoulder ${\mathrm{Fe}}_{\mathrm{sh}}^{2+}$ at ∼708.4 eV are determined in dependence of Ni content x in relation to the total area of the Fe 2$p_{3/2}$ spectrum A(Fe 2$p_{3/2}$) [cf. Figure 7d]. The intensity of the ${\mathrm{Fe}}^{3+}$ satellite is only slightly increasing for low but increasing Ni contents x, whereas its intensity is significantly increasing when surpassing the stoichiometric Ni content of x = 1 and almost constant for further increased Ni content x. In contrast, the intensity of the low binding energy ${\mathrm{Fe}}^{2+}$ shoulder is continuously decreasing for an increasing Ni content up to x ≈ 1, while it almost vanishes for even higher Ni content. Both observations therefore constitute a decreasing relative amount of ${\mathrm{Fe}}^{2+}$ cations when increasing the Ni content up to x = 1 in the ferrite films, whereas the relative ${\mathrm{Fe}}^{3+}$ amount is increased and constant for x > 1. Further, this behavior is in accordance with the shift to higher binding energies of the Fe 2$p_{3/2}$ and Fe 2$p_{1/2}$ spectra, as demonstrated above.

### 3.5. AR-HAXPES

**Figure 8:**
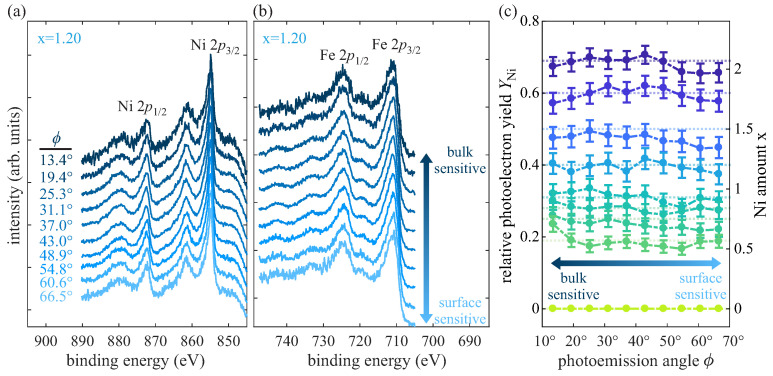
(**a**) Ni 2*p* spectra and (**b**) Fe 2*p* spectra recorded at different off-normal photoemission angles ϕ for the sample with x = 1.20. Low photoelectron emission angles correspond to more bulk-like sensitivity, whereas higher photoelectron emission angles mean higher surface sensitivity. (**c**) Relative photoelectron yields $Y_{\mathrm{Ni}}$ and Ni contents x = $3\cdot Y_{\mathrm{Ni}}$ in dependence of the photoelectron emission angle for films with varying Ni content. For comparison, the respective Ni amounts x as determined by surface sensitive XPS are indicated by dotted lines.

In addition to the investigation of the cationic valencies within the prepared Ni_x_Fe_3−x_O_4_ films, a depth-dependent determination of the cationic stoichiometry is conducted by means of angle resolved detection of Ni 2*p* and Fe 2*p* spectra. Figure 8a,b show the respective AR-HAXPES Ni 2*p* and Fe 2*p* spectra detected at different photoelectron emission angles ϕ exemplarily for the ferrite film with x = 1.20. Lower angles of photoelectron emission correspond to a more bulk-like sensitivity, whereas higher photoelectron emission angles mean higher surface sensitivity. All Ni and Fe 2*p* spectra exhibit no significant deviations in shape and position among each other in dependence of the detection angle. Thus, the cationic valencies (as described above) do not change with depth. This finding points to a uniform distribution of the determined cationic oxidation states in vertical direction.

Further, a depth-dependent determination of the Ni amount x is performed by calculating the relative photoelectron yield $Y_{\mathrm{Ni}}^{2p}$ [cf. Equation (2)] with the numerically integrated areas $A_{\mathrm{Ni},\mathrm{Fe}}^{2p}$ and taking into account the differential photoelectric cross sections $\sigma_{\mathrm{Ni},\mathrm{Fe}}^{2p}$ from Trzhaskovskaya et al. under consideration of non-dipole parameters of the photoelectron angular distribution [35,36]. Prior to this, a Shirley background has been subtracted from the Ni 2*p* and Fe 2*p* spectra.

With this, the Ni content x = $3\cdot Y_{\mathrm{Ni}}$ for each sample is determined in dependence of the photoelectron emission angle ϕ probing different depths. The results are depicted in Figure 8c along with the calculated values obtained from surface sensitive XPS (dotted lines). The Ni amount as determined by AR-HAXPES analysis shows no significant variations in dependence of the photoemission angle ϕ, indicating uniform vertical distributions of Ni and Fe cations within the films independent of the Ni content x. Further, within the experimental uncertainties the Ni amount x as determined by AR-HAXPES agrees well with the values obtained by surface sensitive XPS for all films. Hence, all films can be considered as exhibiting homogeneous cationic distributions in vertical direction without indicating the existence of layers with separated phases within the films or at the interface/surface.

### 3.6. XAS

**Figure 9:**
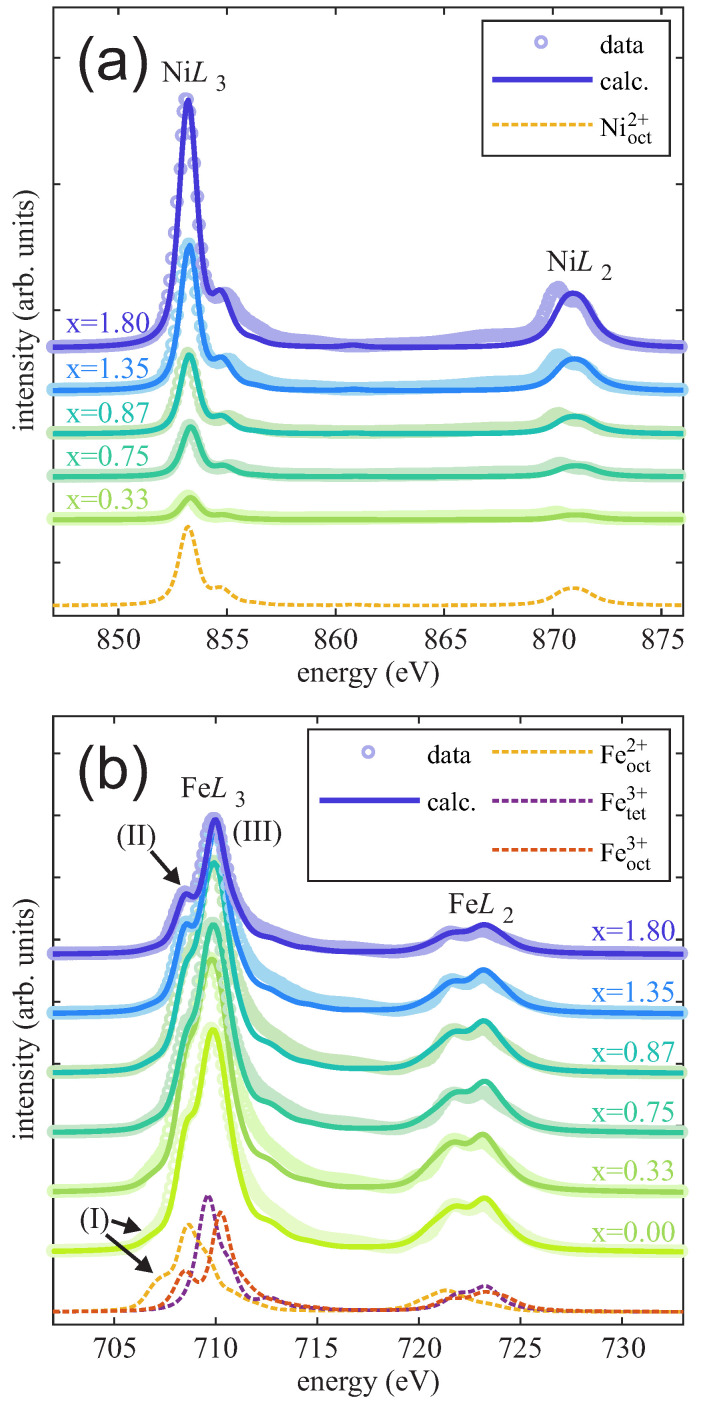
XAS spectra of the ferrite films with varying Ni content x at (**a**) Ni $L_{2,3}$ edges and (**b**) Fe $L_{2,3}$ edges. Dashed lines show single contributions to the XAS spectra using CTM calculations.

Complementary to the HAXPES measurements, XAS measurements were performed to probe the cationic valence states of Ni and Fe cations as well as their lattice site occupancies within the ferrite films. The spectra recorded at the Ni $L_{2,3}$ and Fe $L_{2,3}$ absorption edges for varying Ni content x are shown in Figure 9a and Figure 9b, respectively.

As shown in Figure 9a, the overall intensity of the Ni $L_{2,3}$ XAS spectra increases with increasing Ni content x. However, the shapes of the spectra are independent of the Ni content x and closely resemble reported NiO and NiFe_2_O_4_ X-ray absorption spectra where ${\mathrm{Ni}}_{\mathrm{oct}}^{2+}$ cations are located on B-sublattice sites with octahedral coordination [8,29,37,38,39]. In fact, the measured spectra can be described very well by the sole CTM spectrum calculated for ${\mathrm{Ni}}_{\mathrm{oct}}^{2+}$ cations. Thus, a major presence of only ${\mathrm{Ni}}_{\mathrm{oct}}^{2+}$ cations within the films without any significant variation in the valence state or site occupancy with varying x can be assumed.

In contrast to the intensity increase of the Ni $L_{2,3}$ absorption spectra, the overall intensity of the total Fe $L_{2,3}$ XAS signal decreases with increasing Ni content x due to the decreasing relative Fe content 3−x. Similar to the Ni $L_{2,3}$-edge spectra, all Fe $L_{2,3}$-edge spectra agree very well with the CTM calculations. In addition to the intensity decrease, some variations in the shape of the spectra are observed. First, the pre-edge feature (I) (cf. Figure 9b) at the low energy side of the Fe $L_{3}$ edge (∼707.0 eV) is present for x < 1 but decreases and vanishes for increasing Ni content x > 1. Comparison with the CTM calculated Fe $L_{2,3}$-edge spectra, this feature (I) can be attributed to the presence of octahedrally coordinated ${\mathrm{Fe}}_{\mathrm{oct}}^{2+}$. Its decrease in intensity can therefore be assigned to the decreasing ${\mathrm{Fe}}_{\mathrm{oct}}^{2+}$ content, which is in excellent agreement with the HAXPES results (cf. Section 3.4). Second, with increasing Ni content x, a more distinct feature (II) at ∼708.5 eV becomes distinguishable from the main Fe $L_{3}$-absorption line (III) at ∼709.9 eV. The fact that this feature (II) is clearly visible for x > 1 and not smeared out with the main Fe $L_{3}$ line also indicates the loss of ${\mathrm{Fe}}_{\mathrm{oct}}^{2+}$, as the ${\mathrm{Fe}}_{\mathrm{oct}}^{2+}$ absorption spectrum would add significant intensity between lines (II) and (III) and result in a broader smeared out main Fe $L_{3}$ absorption line (cf. spectra presented above for Ni content x < 1) as demonstrated by the calculated CTM spectra shown in Figure 9b (dashed lines).

**Figure 10:**
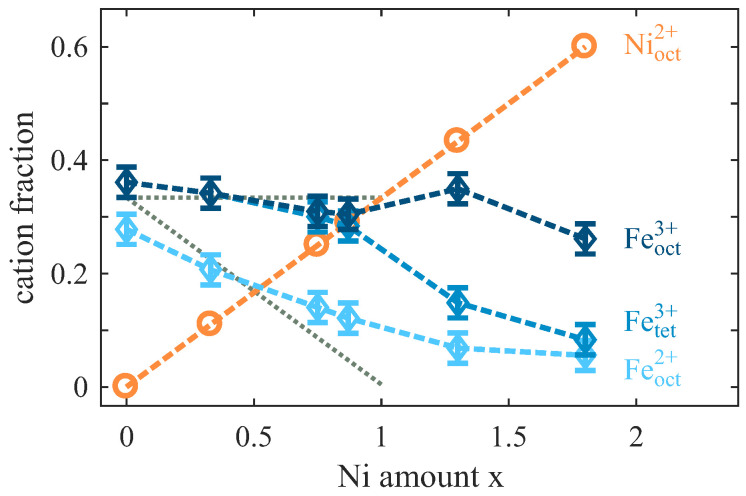
Fraction of the different cations for varying Ni content x in the ferrite films. The Ni fraction and the gross Fe fraction are obtained from XPS while additional CTM calculation based analysis of the XA Fe $L_{2,3}$-edge spectra is used to obtain the fraction of the individual Fe cations with different oxidation state and coordination. The grey dotted lines show the expected fractions of the different Fe cations assuming that ${\mathrm{Fe}}_{\mathrm{oct}}^{2+}$ are gradually substituted by ${\mathrm{Ni}}_{\mathrm{oct}}^{2+}$ in Ni_x_Fe_3−x_O_4_ for increasing Ni content x.

The ratio of the resulting single cationic CTM calculated spectra contributing to the experimental spectra provide the relative fraction of each cation considering oxidation state and coordination in the respective to lattice site occupancy as shown in Figure 10. For the magnetite film (x = 0), a slightly lower ${\mathrm{Fe}}_{\mathrm{oct}}^{2+}$ content in relation to the similar ${\mathrm{Fe}}_{\mathrm{oct}}^{3+}$ and ${\mathrm{Fe}}_{\mathrm{tet}}^{3+}$ contributions is observed, which at first sight contradicts stoichiometric and complete inverse spinel Fe_3_O_4_. However, as indicated by the surface cation vacancy (SCV) model for the reconstructed $\left(\sqrt{2}\times \sqrt{2}\right)R45^{\circ }$ Fe_3_O_4_(001) surface, well-ordered Fe_3_O_4_(001) exhibits a slight excess of ${\mathrm{Fe}}^{3+}$ in the topmost layers followed by stoichiometric ${\mathrm{Fe}}^{2+}$:${\mathrm{Fe}}^{3+}$ ratios in the layers lying underneath [40,41]. As the XAS measurements were conducted in surface sensitive TEY mode, topmost layers contribute more to the XAS signal than to deeper layers. Thus, the observed enrichment of ${\mathrm{Fe}}^{3+}$ in the XAS data of the magnetite film is still consistent with stoichiometric Fe_3_O_4_, taking into account the SCV model for the reconstructed Fe_3_O_4_(001) surface.

With increasing Ni content x, the fraction of ${\mathrm{Fe}}_{\mathrm{oct}}^{2+}$ cations continuously decreases for x < 1, whereas the number of ${\mathrm{Fe}}^{3+}$ cations almost remains constant with about one cation/f.u. (fraction 1/3) on tetra- and octehedral sites, respectively. This behavior is consistent with the successive replacement of ${\mathrm{Fe}}_{\mathrm{oct}}^{2+}$ with ${\mathrm{Ni}}_{\mathrm{oct}}^{2+}$ cations for x ≤ 1 as depicted by dotted lines in Figure 10. For x > 1, the amount of ${\mathrm{Fe}}_{\mathrm{oct}}^{2+}$ cations attains its minimum with ∼0.17 cations/f.u. (fraction 0.06) at x = 1.80. Moreover, the ${\mathrm{Fe}}_{\mathrm{tet}}^{3+}$ crucially decreases after passing the stoichiometric Ni content of x = 1, while the ${\mathrm{Fe}}_{\mathrm{oct}}^{3+}$ content almost remains constant with only a slight decrease for x = 1.80. This observation indicates the decreasing contribution of inverse spinel-type structures within the NFO films with x > 1 due to the decreasing occupancy of tetrahedral sites.

### 3.7. SQUID

**Figure 11:**
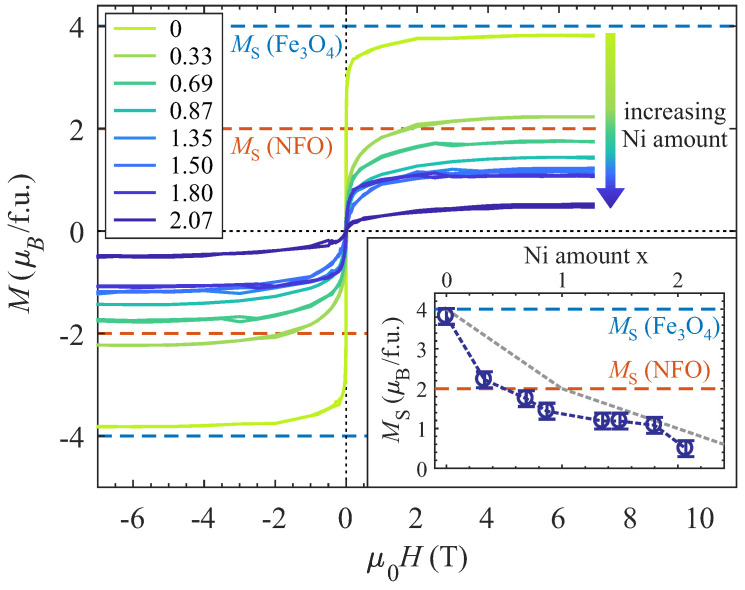
*M* vs. *H* measurements for Ni_x_Fe_3−x_O_4_ ultrathin films with varying Ni content x measured by SQUID magnetometry. The saturation magnetization $M_{S}$ is decreasing for increasing Ni content (see inset). The $M_{S}$ values for stoichiometric Fe_3_O_4_ and NiFe_2_O_4_ (NFO) are depicted for comparison (dashed horizontal lines). If ${\mathrm{Ni}}^{2+}$ cations ideally substitute ${\mathrm{Fe}}^{2+}$ for x ≤ 1 on octahedral sites and further ${\mathrm{Ni}}^{2+}$ cations in the overstoichiometric regime x > 1 also solely occupy octahedral sites, some decrease is expected as indicated by the grey dashed line in the inset.

Magnetic characterization of the Ni_x_Fe_3−x_O_4_ films with varying Ni content x was performed by applying SQUID magnetometry. Therefore, *M* vs. *H* measurements with a maximum applied magnetic field of ±7 T oriented parallel to the sample surface in [100] direction (according to the magnetic easy axis of Fe_3_O_4_ and NiFe_2_O_4_) of substrate and film (in-plane geometry) were conducted at 300 K sample temperature. To determine the magnetic response solely of the Ni_x_Fe_3−x_O_4_ film, a linear background stemming from the MgO substrate and sampel holder was subtracted from the data. The resulting *M* vs. *H* curves are depicted in Figure 11.

All magnetization curves exhibit hysteretic behavior, which is characteristic for ferro-/ferrimagnetic material, with magnetic saturation reached at an applied magnetic field above ∼5 T. However, the coercive fields $H_{C}$ (where the magnetization vanishes) is very small with values between 5 ± 2 and 20 ± 2 mT without showing any trend in dependence of the Ni content x.

In contrast, the saturation magnetization $M_{S}$ of the ferrite films exhibits a continuous decrease with increasing Ni content x (cf. inset of Figure 11). For the Fe_3_O_4_ film (x = 0), the saturation magnetization almost matches the bulk magnetization of stoichiometric Fe_3_O_4_ of 4 $\mu_{B}$/f.u. (if only spin magnetic moments are considered and small orbital magnetic moments are neglected due to orbital quenching). However, already for x ≥ 0.87, the saturation magnetization $M_{S}$ drops below the expected value of 2 $\mu_{B}$/f.u. for stoichiometric bulk and complete inverse spinel NiFe_2_O_4_. A further increase of Ni content x also results in a further decrease in the saturation magnetization $M_{S}$ down to a value of 0.5 $\mu_{B}$/f.u. for the film with x = 2.07. If ideal substitution of ${\mathrm{Fe}}^{2+}$ by ${\mathrm{Ni}}^{2+}$ cations on octahedral sites is considered within the understoichiometric regime (x ≤ 1) a linear decrease from 4 $\mu_{B}$/f.u. for x = 0 to 2 $\mu_{B}$/f.u. for x = 1 would be expected (grey dashed line in the inset of Figure 11). If further incorporation of ${\mathrm{Ni}}^{2+}$ cations in the overstoichiometric regime (x > 1) solely takes place on octahedral sites, which would give rise to the formation of rock salt like coordination, and assuming only antiferromagnetic coupling in these rock salt type structures, a further decrease with a vanishing saturation magnetization $M_{S}$ should be expected (grey dashed line in the inset of Figure 11). Though, the determined values within both regimes remain below these theoretical predictions and point to some degree of disorder.

## 4. Discussion

As revealed by HAXPES, the overstoichiometric Ni_x_Fe_3−x_O_4_ films with x > 1 exhibit almost only ${\mathrm{Fe}}^{3+}$ cations, while spectra obtained from understoichiometric films with x < 1 also show contributions due to ${\mathrm{Fe}}^{2+}$ cations. On the one hand, this result agrees well with the assumption that ${\mathrm{Fe}}^{2+}$ cations are gradually substituted by ${\mathrm{Ni}}^{2+}$ cations for increasing Ni content. On the other hand, the rising high binding energy shoulder as well as the altered satellite structure and the shift to lower binding energies of the Ni 2$p_{3/2}$ photoemission spectrum for overstoichiometric films with x > 1 show that an increasing fraction of the ${\mathrm{Ni}}^{2+}$ cations is located in the NiO rocksalt configuration instead of the NFO inverse spinel configuration.

This (qualitative) result can be quantified better analyzing XAS data by CTM calculations. The analysis provides an enhanced ${\mathrm{Fe}}^{3+}$:${\mathrm{Fe}}^{2+}$ ratio compared to the understoichiometric regime. However, a small amount of octahedrally coordinated ${\mathrm{Fe}}_{\mathrm{oct}}^{2+}$ is still observed. More remarkably, also a crucial decrease in tetrahedrally coordinated ${\mathrm{Fe}}_{\mathrm{tet}}^{3+}$ is determined for x > 1, which can be assigned to a decreasing amount of inverse spinel-type structures in the overstoichiometric NFO films.

**Figure 12:**
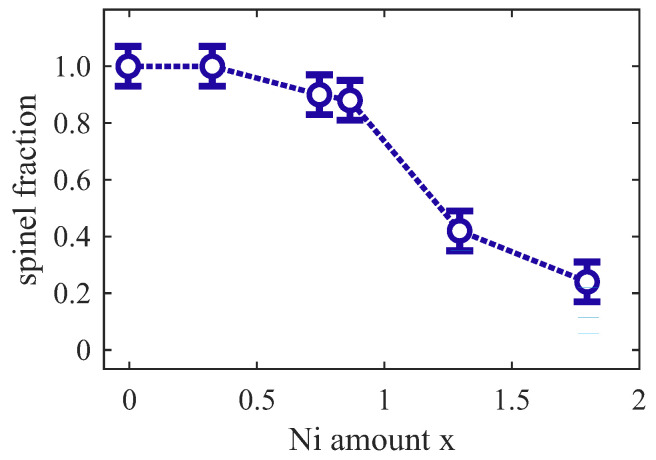
Spinel fraction of Ni-Fe oxide film identified by the fraction of tetrahedrally coordinated ${\mathrm{Fe}}^{3+}$ cations.

Figure 12 shows the fraction of the oxide film assuming that ${\mathrm{Fe}}_{\mathrm{tet}}^{3+}$ can be identified with the film fraction with an inverse spinel structure. Obviously, films have an inverse spinel structure for low Ni contents x≪1. For x≲1, however, there is an excess of octahedrally coordinated cations that cannot all be incorporated into the inverse spinel part of the film. Since these cations are octahedrally coordinated, it can be concluded that they are incorporated in some rocksalt-like structure, which may be of the type ${\mathrm{Fe}}_{y}$${\mathrm{Ni}}_{1-y}$O (Fe-doped NiO). It has to be emphasized that the fraction of ${\mathrm{Fe}}_{\mathrm{oct}}^{2+}$ cations and ${\mathrm{Ni}}_{\mathrm{oct}}^{2+}$ cations incorporated in the inverse spinel structure or in the rocksalt structure cannot be determined.

For x > 1, the major parts of the films have a rocksalt structure and the inverse spinel part is strongly suppressed. In addition, there is also an excess of octahedrally coordinated ${\mathrm{Fe}}_{\mathrm{oct}}^{3+}$ cations compared to the fraction of tetrahedrally coordinated ${\mathrm{Fe}}_{\mathrm{tet}}^{3+}$ (cf. Figure 10). Thus, there have to be cation vacancies in the rocksalt part of the film due to charge neutrality. Therefore, this deficient rocksalt structure may be denoted by ${\mathrm{Fe}}_{y}$${\mathrm{Ni}}_{1-y}$$O_{1+\delta }$ with oxygen excess δ as discussed for cation ordering in Fe_3_O_4_ [42].

XRR and AR-HAXPES studies, however, give no indications of layers of additional or separated phases neither in the conversion from Fe_3_O_4_ to NiFe_2_O_4_ for x < 1 nor from NiFe_2_O_4_ to an NiO-like rocksalt phase for x > 1. In fact, both measurement techniques point to single crystalline films with homogeneous cation and valence state depth distributions. Thus, in particular, both inverse spinel parts as well as rocksalt parts are randomly distributed within the ferrite film without forming distinctly separated layers. The latter has been reported by Kuschel et al., where NiFe_2_O_4_ films were prepared by the interdiffusion of Fe_3_O_4_/NiO bilayers on ${\mathrm{SrTiO}}_{3}$(001) induced by post-deposition annealing (PDA) [32]. The final film stack of the PDA treatment exhibits a segregated NiO layer on top of the NiFe_2_O_4_ film, when the initial Ni:Fe ratio exceeds the stoichiometric ratio of 1:2. Note that the same behavior is noticed for similar PDA treatment of Fe_3_O_4_/CoO bilayers, where an ultrathin CoO layer segregates to the top of the formed CoFe_2_O_4_ film [43]. As a consequence, the co-evaporation method as performed in this study suppresses the NiO segregation to the surface due to the significantly lower sample temperature of 250 °C during film growth compared to at least 600 °C for the alternate PDA preparation method.

The finding of single crystalline films with homogeneous cationic distributions throughout the whole range of Ni content x is confirmed by the observation of only single diffraction peaks in HR-XRD measurements. Two strictly separated but coexisting phases, e.g., agglomeration in inverse spinel and rocksalt clusters with different lattice constants, would also result in different (vertical) layer distances and, therefore, in the observation of separated diffraction peaks, if the crystalline regions of each phase are sufficiently large. Finally, the Ni-Fe oxide film has an alloy-like structure mixing the locally inverse spinel and rocksalt structures without long-range order. In addition, the cation distribution in these different local structures is undetermined, and the overall structure may be denoted by [Ni_x_Fe_3−*x*_O_4_]_1−*z*_ [${\mathrm{Fe}}_{y}$${\mathrm{Ni}}_{1-y}$$O_{1+\delta }$]_*z*_.

The in situ LEED measurements on the surface structure of the ultrathin Ni_x_Fe_3−x_O_4_ films reveal structural changes with varying Ni content x of the films. First, the diffraction pattern of an inverse spinel surface with a $\left(\sqrt{2}\times \sqrt{2}\right)R45^{\circ }$ superstructure is observed for Ni content x ≪ 1. This superstructure is characteristic for Fe_3_O_4_(001) surfaces and can be observed up to a Ni contents x ≤ 0.7.

For intermediate Ni contents x ≲ 1, the superstructure vanishes, but the fundamental (1×1)_S_ spinel pattern remains. This pattern is characteristic for NiFe_2_O_4_(001). This behavior comes along with a decreasing vertical layer distance as determined by HR-XRD analysis, which is also expected considering the decrease in bulk layer distances from Fe_3_O_4_ to NiFe_2_O_4_. These observations made in LEED and XRD therefore indicate a subsequent conversion from Fe_3_O_4_ to NiFe_2_O_4_ when increasing the Ni content x fro, x = 0 to x = 1. Further, within this conversion for x < 1, HAXPES measurements reveal a decrease in the relative ${\mathrm{Fe}}^{2+}$ content accompanied by an increase in the relative ${\mathrm{Fe}}^{3+}$ amount within the Fe cations. However, the ${\mathrm{Ni}}^{2+}$ valency remains unchanged, which indicates that the increase in the Ni content can be associated with the expected (but incomplete) exchange of ${\mathrm{Fe}}^{2+}$ by ${\mathrm{Ni}}^{2+}$ cations. This replacement with increasing x is reinforced by the quantitative analysis of XAS spectra by means of CTM calculations (see above).

For intermediate Ni contents x ≳ 1, the apparent fundamental (1×1)_S_ spinel pattern can be observed although XAS analysis points to an increasing fraction of local rocksalt structures. However, the existing rocksalt-related LEED pattern may be hidden by the spinel-related LEED pattern since the reciprocal surface unit cell of the latter has half the size of the reciprocal rocksalt unit cell. Clear conversion of the surface structure from inverse spinel to rocksalt is observed for x > 1.5.

**Figure 13:**
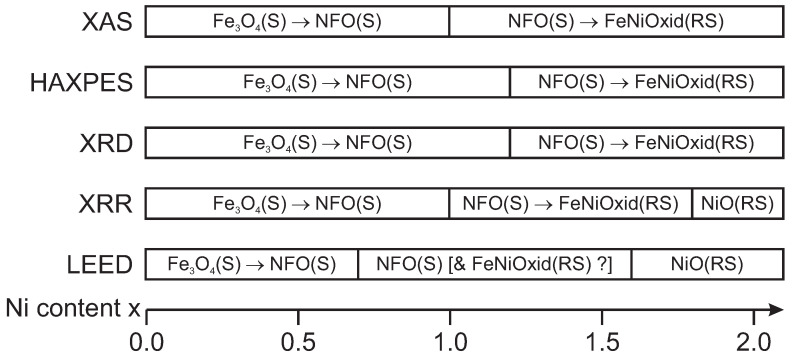
Overview of different phases of ultrathin Ni_x_Fe_3−x_O_4_ film as determined by different experimental techniques depending on Ni content x.

Depending on Ni content x, Figure 13 presents an overview of different phases of ultrathin Ni_x_Fe_3−x_O_4_ films as determined by different experimental techniques.

This transition is in accordance with the subsequent increase in the vertical layer distance when increasing x in the overstoichiometric regime as determined by HR-XRD. This is also according to the slight increase when comparing the bulk layer distances of NiFe_2_O_4_ and NiO. In addition, due to the larger bulk NiO layer distance compared to the one of NiFe_2_O_4_, NiO exhibits a smaller lattice mismatch to the MgO substrate. This should also result in smaller vertical compressive strain, which could further explain the increased layer distances for increasing x in the overstoichiometric regime. Moreover, a significant decrease in the interface distance for x > 1.20 as determined by HR-XRD analysis also points to the formation of a different structure at the interface. However, the oxide film surface is still extremely smooth, and its roughness is only slightly increased for the overstoichiometric films compared to the understoichiometric regime.

The out-of-plane lattice constants obtained by HR-XRD are all compressively strained as can be explained by an at least partial adaption of the film to the substrate lattice (pseudomorphic growth mode) resulting in lateral expansion and vertical compression. Based on elastic theory for the continuum and implying a homogeneous tetragonally strained film structure due to in-plane tensile stress, the expected vertical layer distance can be estimated by

(3) $$\frac{\Delta d_{\mathrm{vert}}}{d_{\mathrm{vert}}}=\frac{\Delta c}{c}=\frac{2\nu }{\nu -1}\frac{\Delta a}{a}$$

with ν as the Poisson ratio of the film material [44]. Here, *c* and *a* are the bulk vertical and lateral lattice constants, whereas Δc and Δa denote the respective differences between strained and bulk lattice constants. Following Equation (3) and taking the Poisson ratios for the stoichiometric cases of Fe_3_O_4_ ($\nu_{{\mathrm{Fe}}_{3}O_{4}}$ = 0.356 [45]), NiFe_2_O_4_ ($\nu_{\mathrm{NFO}}$ = 0.338 [45]), and NiO ($\nu_{\mathrm{NiO}}$ = 0.297 [45]) into account, compressed vertical layer distances of $d_{\mathrm{vert}}^{{\mathrm{Fe}}_{3}O_{4}}$ = 209.1 pm, $d_{\mathrm{vert}}^{\mathrm{NFO}}$ = 206.3 pm, and $d_{\mathrm{vert}}^{\mathrm{NiO}}$ = 207.3 pm are obtained for the three respective stoichiometric oxides, assuming complete pseudomorphic adaption of the lateral lattice constants to the lattice constant of the MgO substrate. These estimated values [according to the lower limits of the filled regions in Figure 5a] match very well with the evolution of the measured strained vertical layer distances, which in turn confirms complete adaption of the film to the substrate lattice without any hint of strain relaxation. As a consequence thereof, all prepared ferrite films are assumed to exhibit negligible amounts of defects like misfit dislocations, which would release the applied strain. This assumption is confirmed by the complete vertical crystallinity and high crystalline quality of the ferrite films with very smooth interfaces/surfaces independent of the Ni content as determined by HR-XRD analysis.

Further, the assumed absence of strain releasing defects can be followed by estimating the theoretical critical thickness D$D_{c}$ above which misfit dislocations are incorporated into the film to release strain. Using the model of Matthews and Blakeslee [46], the critical thickness $D_{c}$ for the initial cooperation of dislocations is given by

(4) $$\frac{D_{c}}{b}=\frac{\left(1-\nu \;\cos^{2}\alpha \right)\;\left(\ln\left(\frac{D_{c}}{b}\right)+1\right)}{2\;\pi \;f\;(1+\nu )\;\cos(\lambda )}.$$

Here, *b* is the magnitude of the Burgers vector, *f* is the modulus of the lattice mismatch, α = 90° is the angle between the Burgers vector and the dislocation line, and λ = 45° is the angle between the Burgers vector and the direction normal to the dislocation line and within the plane of the interface. For Fe_3_O_4_ and NiFe_2_O_4_, the magnitudes of the Burgers vectors for the different involved oxides are $b_{{\mathrm{Fe}}_{3}O_{4}}=a_{0}^{{\mathrm{Fe}}_{3}O_{4}}$/4 = 296.8 pm and $b_{\mathrm{NFO}}=a_{0}^{{\mathrm{NiFe}}_{2}O_{4}}$/4 = 294.8 pm ($a_{0}$: cubic bulk lattice constants) [47]. For NiO, the magnitude of the Burgers vector is given by $b_{\mathrm{NiO}}=a_{0}^{\mathrm{NiO}}$/2 = 295.3 pm. Taking the respective Poisson ratios into account (see above), critical thicknesses of 103.9 nm, 27.4 nm, and 34.8 nm are obtained for Fe_3_O_4_, NiFe_2_O_4_, and NiO, respectively. All values are significantly larger than the prepared film thicknesses within this study (up to 18.6 nm) and are therefore consistent with the observed homogeneous strain. Moreover, this observation agrees with the results obtained for the off-stoichiometric NFO films in a former study [48].

Apart from the structural and electronic characterization, magnetization *M* vs. *H* measurements reveal a decrease in saturation magnetizations $M_{S}$ as it is expected for increasing Ni content x. For x = 0 (Fe_3_O_4_), the measured saturation magnetization matches the expected value of 4 $\mu_{B}$/f.u. with only a slight deviation to a lower value. The subsequent decrease in $M_{S}$ for increasing x is stronger than expected for the gradual substitution of ${\mathrm{Fe}}_{\mathrm{oct}}^{2+}$ cations by ${\mathrm{Ni}}_{\mathrm{oct}}^{2+}$ cations, causing the magnetization of 2 $\mu_{B}$/f.u. for stoichiometric bulk NiFe_2_O_4_ (x = 1). Here, the saturation magnetization drops below the value of 2 $\mu_{B}$/f.u. already for Ni content x ≥ 0.87.

Partly, this low saturation magnetization may be due to the coexistence of a ferrimagnetic NFO spinel structure with antiferromagnetic rocksalt structures. However, major parts of this deviation could be ascribed to the presence of APBs in the ferrite films [49], which are typically formed during the film growth of the ferrite with a double-sized lattice constant compared to the one of the MgO substrate. This discrepancy forces that the islands or layers formed during film growth with nucleation centers that are non-integer multiples of the ferrite lattice constant apart from each other are out-of-phase upon merging and, thus, form APBs. Strong antiferromagnetic coupling across the APBs as it is known for, for example, Fe_3_O_4_ on MgO(001) [50], could consequently result in a lower saturation magnetization as it is observed here for all prepared ferrite films.

Further, the observed discrepancy to lower $M_{S}$ values for x > 1 could be ascribed to the decreasing ${\mathrm{Fe}}_{\mathrm{tet}}^{3+}$:${\mathrm{Fe}}_{\mathrm{oct}}^{3+}$ ratio and the consequently decreasing content of inverse spinel-type structures. The relative ${\mathrm{Fe}}_{\mathrm{oct}}^{3+}$ excess compared to the spinel-type structures could possibly couple antiferromagnetically in the rock-salt-like structures and would therefore not give a ferromagnetic contribution to the magnetization and give rise to an enhanced discrepancy of $M_{S}$ to lower values.

## 5. Conclusions

Within this study, a structural, chemical, and magnetic characterization was conducted on Ni_x_Fe_3−x_O_4_ ultrathin films with thickness 16 ± 2 nm and varying Ni content x. The films were prepared by RMBE on lattice-matched MgO(001). All films are compressively strained in the vertical direction due to lateral tensile strain, resulting from an adaption of the film to the substrate lattice. As a consequence, the films are assumed to exhibit very low defect densities (except for APBs, see below) as the results and theoretical predictions show no evidence of released strain by defects like, for example, misfit dislocations. The films are completely crystalline from the interface to the surface with extremely low interface and surface roughnesses, independent of the cationic stoichiometry.

On the one hand, for understoichiometric Ni content x < 1, a successive conversion from Fe_3_O_4_ to (almost) stoichiometric NiFe_2_O_4_ with increasing x is observed while conserving the inverse spinel structure. Within this regime, octahedrally coordinated ${\mathrm{Fe}}_{\mathrm{oct}}^{2+}$ cations are replaced by ${\mathrm{Ni}}_{\mathrm{oct}}^{2+}$ cations, according to the decreasing ${\mathrm{Fe}}^{2+}$:${\mathrm{Fe}}^{3+}$ ratio and a constant ${\mathrm{Ni}}^{2+}$ valency as observed in HAXPES and XAS. On the other hand, for the overstoichiometric regime x > 1, a crucial decrease in tetrahedrally coordinated ${\mathrm{Fe}}_{\mathrm{tet}}^{3+}$ cations (attributed to the inverse spinel structure of NFO) is noticed and mostly ${\mathrm{Fe}}_{\mathrm{oct}}^{3+}$ and ${\mathrm{Ni}}_{\mathrm{oct}}^{2+}$ cations are present within the films, indicating a decrease in inverse spinel-type structures. Parts of the films show a local rocksalt structure for mixed Fe-Ni monoxide. However, these parts are homogeneously distributed within the films and not strictly separated from the ferrite-like NFO parts.

The saturation magnetization of the films decreases, converting Fe_3_O_4_ to NiFe_2_O_4_. However, the decrease is stronger than expected for this conversion. This result can be related to the presence of APBs, whereas the discrepancy in the overstoichiometric regime (x > 1) can be related to the presence of antiferromagnetic rocksalt structures.

Apart from the presence of APBs, all films exhibit high crystalline quality with low defect densities and sharp interfaces and surfaces, which is crucial for the implementation in spin filter applications [8,10]. Hence, from the results in the presented study, high applicability in spintronics not only for stoichiometric NiFe_2_O_4_ but also for NFO films with off-stoichiometric cationic ratios could be expected.

## Figures and Tables

**Table 1:** Values for the IMFP λ and information depth $D_{I}^{95}$ in HAXPES measurements ($E_{\mathrm{ph}}$ = 6 keV) for photoelectrons originating from Fe 2*p* and Ni 2*p* orbitals passing through NiFe_2_O_4_ at emission angles $\phi =0^{\circ }$ and $\phi =70^{\circ }$ with respect to the surface normal.

	λ	$D_{I}^{95}\left(\phi =0^{\circ }\right)$	$D_{I}^{95}\left(\phi =70^{\circ }\right)$
Fe 2*p*	7.5 nm	22.5 nm	7.7 nm
Ni 2*p*	7.3 nm	21.9 nm	7.5 nm

**Table 2:** Crystal field energies 10Dq, initial, and final charge transfer energies $\Delta_{\mathrm{init}}$ and $\Delta_{\mathrm{final}}$ for the respective cations in a given ligand field used for the CTM calculations to analyze XAS data.

Cation	10Dq (eV)	$\Delta_{\mathrm{init}}$ (eV)	$\Delta_{\mathrm{final}}$ (eV)
${\mathrm{Fe}}_{\mathrm{oct}}^{2+}$	1.15	6.0	5.0
${\mathrm{Fe}}_{\mathrm{oct}}^{3+}$	1.2	4.0	3.0
${\mathrm{Fe}}_{\mathrm{tet}}^{3+}$	−0.6	4.0	3.0
${\mathrm{Ni}}_{\mathrm{oct}}^{2+}$	1.5	6.0	5.0

## Data Availability

The data presented in this study are available on reasonable request from the corresponding author.
